# A New Method of Establishing Orthotopic Bladder Transplantable Tumor in Mice

**DOI:** 10.7497/j.issn.2095-3941.2012.04.007

**Published:** 2012-12

**Authors:** Xi-hua Yang, Lian-sheng Ren, Guo-ping Wang, Li-li Zhao, Hong Zhang, Zhen-guo Mi, Xihua Bai

**Affiliations:** 1 Department of Comparative Medicine, Shanxi Cancer Institute, Taiyuan 030013, China; 2 Shanxi Institute for Food and Drug Control, Taiyuan 030001, China

**Keywords:** mice, bladder tumor, model

## Abstract

**Objective:**

The present study aims to find a convenient, rapid, and stable method to establish bladder tumor in mice.

**Methods:**

Female Balb/C-nu-nu nude mice (or female T739 mice) were narcotized by sodium pentobarbital at a dosage of 60 mg/kg. The stylet of the 24# venous retention needles was bent in a 5° to 7° angle at a distance of 15 mm from the needlepoint to form a circle with 2.61 mm to 3.66 mm radius when the stylet is rotated. The pipe casing was lubricated with liquid paraffin, and inserted into the bladder cavity. The drift angle stylet was inserted into the pipe casing slowly, rotated for five times, and then pulled out. A cell suspension (0.1 mL) of approximately 1×10^6^ T24 cells (or BTT cells) was then injected immediately.

**Results:**

A total of 60 T739 mice and 60 Balb/C-nu-nu nude mice were inoculated with BTT cells and T24 cells, respectively. The bladder tumor incidence and the average survival time of the tumor-bearing mice were 100% and (26.69±9.24) d and 100% and (34.59±9.8) d for the T739 mice and Balb/C-nu-nu nude mice, respectively.

**Conclusions:**

Using the drift angle stylet to injure the mucous membrane of the urinary bladder can establish a stable bladder transplantable tumor model in mice.

## Introduction

Bladder cancer is one of the most common malignant tumors. Most cases progress to high-grade invasive cancer despite long-standing intravesical therapies. Novel therapeutic treatment options are urgently needed to improve the overall treatment success rates for localized bladder cancer^[^^1^^]^. Therefore, stable, reliable, simple, and reproducible orthotopic animal models are critically important. Suitable animal models provide an opportunity to study the mechanism of pathogenesis and allow the research and development of novel therapeutic agents.

In this study, we have successfully established a model of orthotopic bladder tumor in mice using a drift angle stylet to injure the mucous membrane of the urinary bladder. The tumor cells grew on the wall of the urinary bladder after tumor cell injection. This method is convenient, rapid, and stable for establishing bladder tumor in mice and demonstrates the growth, infiltration, and metastasis of the tumor.

## Materials and Methods

### Preparation of the drift angle stylet

**Figure 1:**
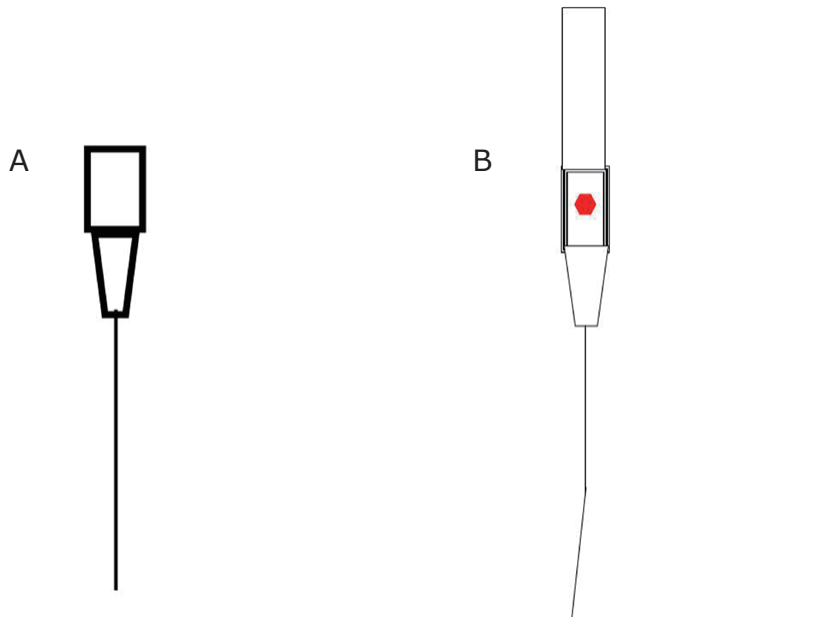
Schematic diagram of the pipe casing (A) and the drift angle stylet (B).

The stylet of the 24# venous retention needles (Becton Dickinson Infusion Therapy Systems, Inc., America) was bent in a 5° to 7° angle at a distance of 15 mm from the needlepoint to form a Φ=2.61 mm to 3.66 mm circle when the stylet was rotated (**Figure 1**).

### Cell strain

Mice urinary bladder transitional cancer cell line BTT-T739, which originated from inbred line T739 mice, is a carcinogen-induced [N-butyl-N-(4-hydroxybutyl) nitrosamine] poorly differentiated transitional carcinoma. The cell line was provided by Professor Yang Xiaofeng from Shanxi Medical University. Human urinary bladder T24 cell strain was purchased from Shanghai Institutes for Biological Science, CAS. The cells were cultured in RPMI 1640 medium supplemented with 10% FBS, 100 U/mL penicillin, and 100 µg/mL streptomycin at 37°C and 5% CO_2_. The cells were harvested by trypsin/EDTA treatment. Viability was determined using the trypan blue exclusion method. The cells were suspended in prepared PBS at a concentration of 1×10^7^/mL.

### Animal

The present study was approved by the local ethics committee and followed the guidelines of the National Research Council Guide for the Care and Use of Laboratory Animals. Female inbred line T739 mice, 4 weeks to 6 weeks old and weighing 20 g to 22 g, were purchased from Beijing HFK Bio-Technology Co. Ltd. The animal produce license number was SCXK (Jing) 2009-0004. Female Balb/C-nu-nu mice, 4 weeks to 6 weeks old and weighing 20 g to 22 g, were purchased from Vital River Laboratories. The animal produce license number was SCXK (Jing) 2006-0009. The animals were bred in the animal laboratory (license number of SYXK (Jin) 2007-0002). The temperature and humidity of the environment was kept at (26±1.5)°C and between 40% and 60%, respectively. The mice were provided with sterilized drinking water and sterile complete nutrition feed.

### The orthotopic transplantation of the tumor cell

**Figure 2:**
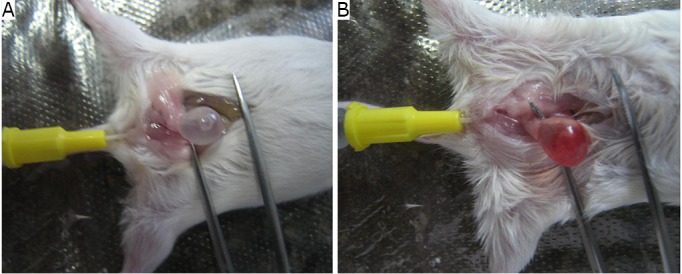
Comparison between the mucosa injured and mucosa uninjured urinary bladder. A: mucosa uninjured; B: mucosa injured. The bladders were injected with physiological saline.

The mice in the experimental group were narcotized by sodium pentobarbital at a dosage of 60 mg/kg. The pipe casing was lubricated with liquid paraffin and inserted into the bladder cavity, and then urine was removed. The drift angle stylet was slowly inserted into the pipe casing. The pipe casing was fixed with one hand, the stylet was rotated for five rounds with the other hand, and then pulled out. Cell suspension (100 µL) of approximately 1×10^6^ cells was injected immediately. The mice in the normal control group were injected with 1×10^6^ cells directly without mucosa injury. The comparison results between the mucosa injured and uninjured urinary bladder are shown in **Figure 2**.

### Observation method

The tumor cell-inoculated mice were fed routinely. The activity and body weight of the mice were observed daily, and survival time was recorded. All mice that died of natural causes were dissected and checked for tumor formation on the urinary bladder and abdominal cavity and hematuria. Metastasis and wet weight of the urinary bladder were recorded after the dissection. The urinary bladder and tumor metastasized organs were fixed in 10% formaldehyde for pathological examination. The observation time of the T739 mice was 40 d, whereas that of the Balb/C-nu-nu mice was 60 d.

## Results

### **Tumor developmen**t

**Figure 3:**
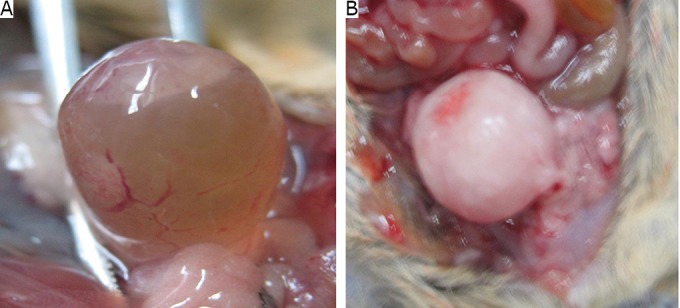
Comparison between the early-stage tumor and advanced-stage tumor on the urinary bladder. A: tumor formation in the early stage; B: tumor formation in the advanced stage.

A total of 60 T739 mice were inoculated with BTT cells in the experimental group. The results showed that the bladder tumor incidence was 100% and the average wet weight of the tumor was (0.54±0.37) g. Ten T739 mice were inoculated with BTT cells in the mucosa uninjured control group; the bladder tumor incidence was 0% (**Table 1****,** **Figure 3**).

**Table 1:** Comparison of tumor incidence between the mucosa uninjured bladder and mucosa injured bladder by drift angle stylet.

Groups	Number of mice	Cell inoculation amount	Tumor developed amount	Tumor incidence (%)
T739 without injury	10	1×10^6^	0	0
T739 with injury	60	1×10^6^	60	100
Balb/C-nu-nu without injury	10	1×10^6^	0	0
Balb/C-nu-nu with injury	60	1×10^6^	60	100

A total of 60 Balb/C-nu-nu nude mice were inoculated with T24 cells in the experimental group. The results showed that the bladder tumor incidence was 100% and the average wet weight was (0.11±0.13) g. The bladder tumor incidence in the normal control group was 0% (**Table 1****,** **Figure 3**).

### Metastasis

**Figure 4:**
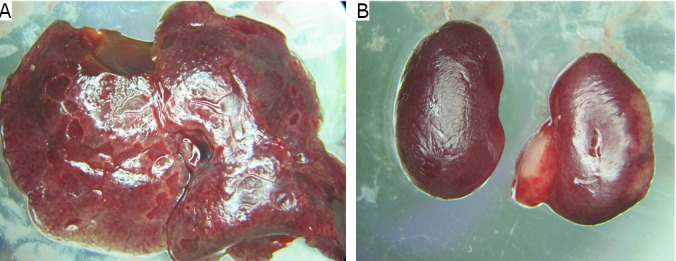
Metastasis in the liver (A) and the kidney (B).

Metastases in the liver, kidney, mesentery, and peritoneum with bloody ascites were observed in 23% of the 60 T739 mice inoculated with BTT cells. Abdominal cavity adhesion was observed, and metastasis occurred either on several viscera (liver, kidney, mesentery, and peritoneum) or only one viscera (kidney) (**Figure 4**). Only one of the 60 Balb/C-nu-nu mice inoculated with T24 cells had metastasis in the kidney (**Figure 4**).

### Survival time

**Figure 5:**
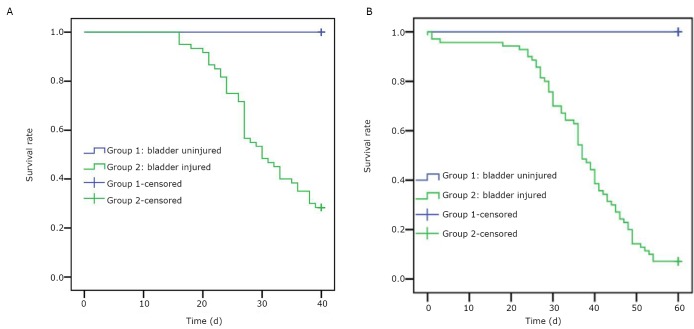
Survival time function of the T739 mice (A) and Balb/C-nu-nu nude mice (B).

T739 mice in the experimental group began to die on the 16th day after BTT cell inoculation. The animals had serious athrepsia with hematuria. The average survival time of the mice was (26.69±9.24) d. In addition, a number of mice died from extreme athrepsia and cachexia (**Table 2**). Balb/C-nu-nu mice in the experimental group began to die on the 18th day after T24 cell inoculation. The average survival time of the mice was (34.59±9.8) d. The mice had extreme athrepsia and cachexia (**Table 2****,** **Figure 5**).

**Table 2:** Average survival time and bladder weight of the mice transplanted with tumor cells.

Groups	Number of mice	Average survival time (days)	Average tumor wet weight
T739 without injury	10	>40	0.02±0.22
T739 with injury	60	26.69±9.24	0.54±0.37
Balb/C-nu-nu without injury	10	>60	0.02±0.32
Balb/C-nu-nu with injury	60	34.59±9.80	0.11±0.13

### Pathological examination

**Figure 6:**
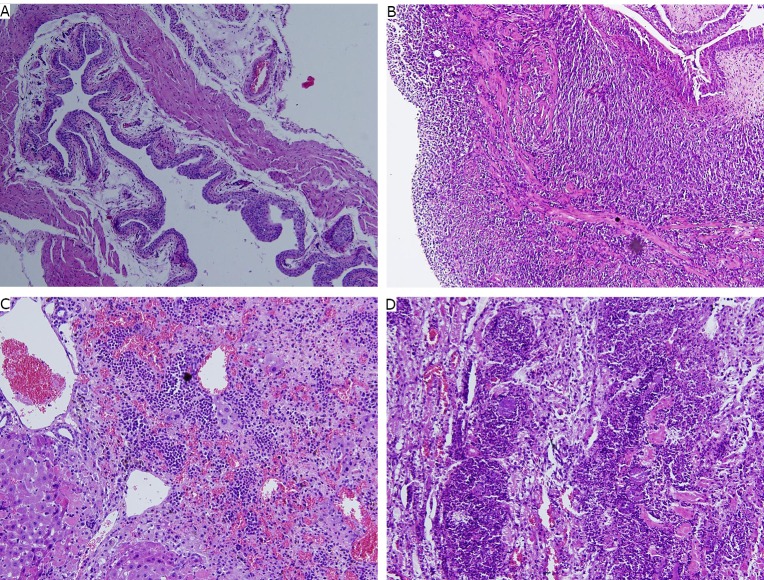
Pathological images of different groups. A: The normal control group bladder (H&E staining, ×100); B: The model group bladder with tumor (H&E staining, ×100); C: The metastasis in liver (H&E staining, ×200); D: The metastasis in kidney (H&E staining, ×200).

The bladder mucosa of the normal control mice was intact, and no metastasis was observed (**Figure 6A**). Huge solid tumors were observed under the microscope. The tumor cells infiltrated deeply into the muscular layer (**Figure 6B**). Cellular atypia was observed in the pathological section of the liver and kidney (**Figures 6C**** and ****6D**).

## Discussion

Orthotopic bladder transplantable tumor in mice is a practical model because it is analogous to the clinical pathological process in humans^[^^1^^]^. Four frequently used methods are classified by their difference in inoculation process, and are described as follows. The first method is intravesical instillation of tumor cell suspension^[^^2^^]^ into denuded bladders, which uses N-methyl-N-nitrosourea^[^^3^^]^ or combination of chlorhydric acid and potassium hydroxide^[^^4^^,^^5^^]^. This method requires no surgery and does not result in diffuse ulceration, edema, or urinary stones. The success rate of tumor implantation ranges from 28% to 97%. Moreover, the tumors appear multifocal and have an unpredictable localization. The second method is injecting tumor cells into the urinary bladder wall through the abdomen^[^^6^^-^^10^^]^. This method requires a skilled surgeon to complete the surgery and is time consuming. The third method is injecting weak acid, protease, or other chemicals into the urinary bladder entocoel to injure the mucous before the injection of tumor cells^[^^4^^,^^11^^-^^13^^]^. This method is also complicated and tumor formation was not stable. The fourth method is the mechanical method. The mucous membrane of the urinary bladder was injured before the injection of tumor cells^[^^14^^]^. Bisson et al.^[^^14^^]^ inserted a stylet with an outside casing into the urinary bladder through the meatus urinarius to injure the mucous. The method does not have any restrictions to the rotation degree of the stylet; thus the degree of injury to the mucous cannot be controlled.

Creating an injury in the mucous of the urinary bladder is important in tumor transplantation. Hemorrhage and damage to the mucous after the injury are beneficial to the adhesion, colonization, and growth of tumor cells. An intact mucosa protects the bladder from tumor cell invasion. The drift angle stylet forms a Φ=2.61 mm to 3.66 mm circle when rotated, causing a balanced injury. Thus, the injury is controllable, and the method is more reasonable.

Bisson et al.^[^^14^^]^ used a custom-made copper abrader to establish a bladder tumor. The result showed that the tumor incidence was approximately 100%. Using a mechanical damage method to induce an injury to the bladder mucosa was feasible to establish an orthotopic bladder tumor. Spotted state injury was induced by friction when rotated. Tiny tumors developed at an early stage and progressed slowly. The drift angle stylet can augment the wound surface when rotated. A number of tumor cells colonized on the mucous at an early stage, and tumors formed within a short period of time.

The present model showed a high metastatic rate. Vesicoureteral reflux may cause renal metastasis. Metastasis may also have a relationship with the cell line because the metastatic rate of T24 was lower than that of BTT. Tumors that has broken through the bladder wall and invaded into the abdominal cavity are in the advanced stage, leading to high metastatic rate.

The 24# venous retention needle used in the present study is a standard medical device and is easy to obtain. The preparation of the drift angle stylet is simple, and the experiment is easy to perform. Moreover, the degree of injury is easy to control.

Using drift angle stylet to injure the mucous membrane of the urinary bladder can establish a stable transplantable bladder tumor model in mice, with convenience and rapidness.
